# Supplementary material for: Northward drift of the Azores plume in the Earth’s mantle
Source: Nat Commun. 2019 Jul 19;10:3235. doi: 10.1038/s41467-019-11127-7 (PMC6642178; doi:10.1038/s41467-019-11127-7)
Supplement: Supplementary file 1 — Supplementary Information [file 41467_2019_11127_MOESM1_ESM.pdf]

Northward drift of the Azores plume in the Earth’s mantle

Maëlis Arnould et al.

Supplementary Table 1 – Numerical modeling parameters.

Parameter	Non-dimensional value	Dimensional value
Surface temperature (T_{top})	0.12	255 K
Basal temperature (T_{bot})	1.12	2450 K
Mantle domain thickness (D)	1	2890 km
Reference thermal expansivity (α)	1	3 x 10^{-5} /K
Reference density (ρ_0)	1	4400 kg/m^3
Reference diffusivity (κ)	1	1 x 10^{-6} m^2/s
Reference conductivity (c_p)	1	3.15 W/(m K)
Reference viscosity (η_0)	1	1.03 x 10^{22} Pa s
Internal heating rate (H)	40	6.81 x 10^{-12} W/kg
Activation energy (E_a)	8	142 kJ/mol
Activation volume (V_a)	3	13.8 cm^3/mol
Maximum viscosity cut-off	10^4	10^{26} Pa s
Viscosity increase at 660 km	30	
Yield stress gradient for all materials ($d\sigma_Y$)	2.34 x 10^6	1088 Pa/m
Yield stress at the surface – oceanic lithosphere (σ_{Yoc})	3.5 x 10^4	48 MPa
Yield stress at the surface – continental interior (σ_{Ycont})	7 x 10^5	932 MPa
Viscosity increase – continental interior	100	
Buoyancy number – continental interior (B_{cont})	-0.32	-150 kg/m^3
Thickness – continental interior	0.0692	200 km
Yield stress at the surface – continental belt (σ_{Ybelt})	3 x 10^5	400 MPa
Viscosity increase – continental belt	50	
Buoyancy number – continental belt (B_{belt})	-0.4	-188 kg/m^3
Thickness – continental belt	0.0432	125 km

Supplementary Figures:

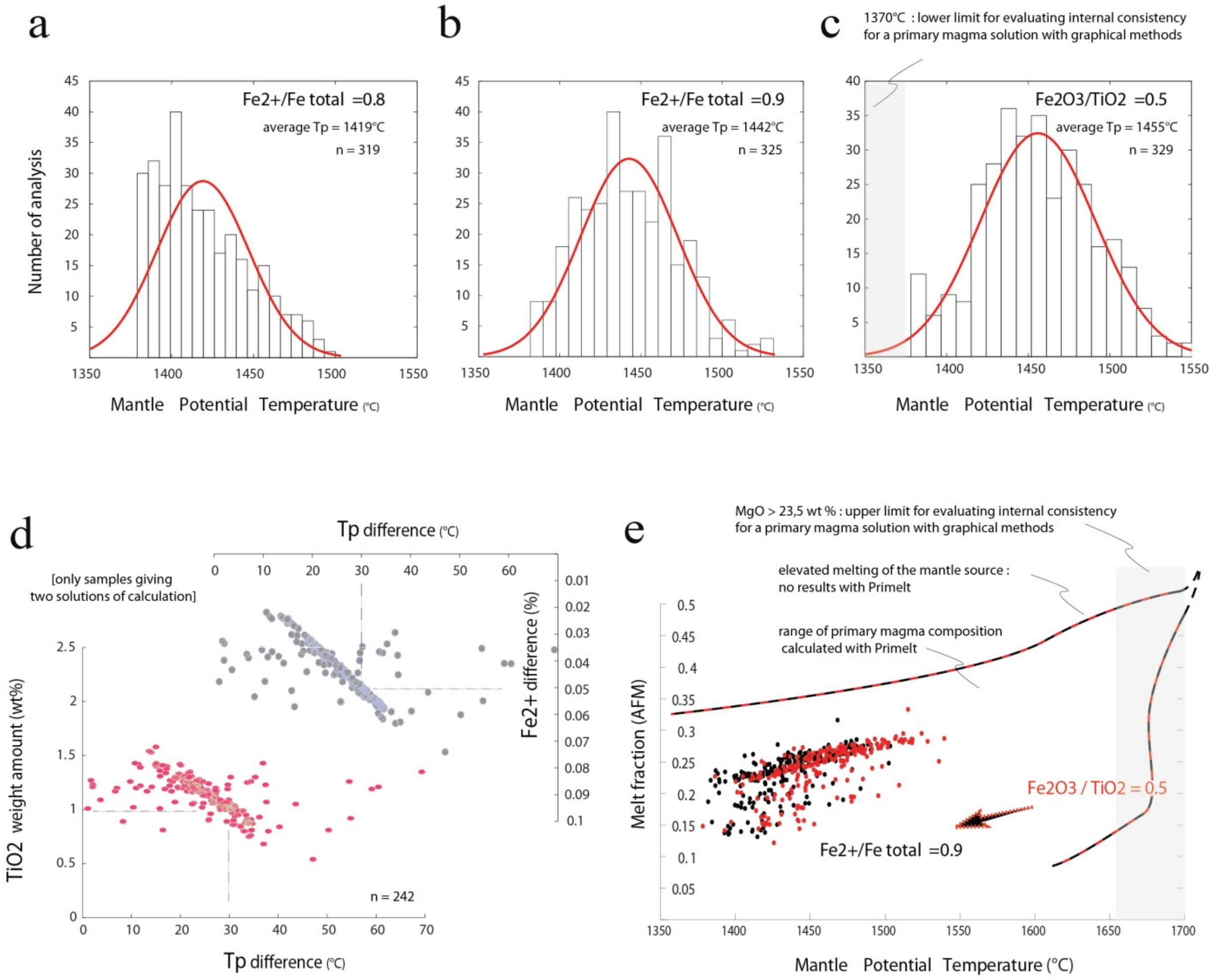

Supplementary Figure 1 - Addressing redox conditions in the magmatic source of the samples of this study[1]. (a, b, c) Comparative distribution (histograms) of potential temperature (T_P) of mantle depicted from the composition of MORBs using PRIMELT3 MEGA software[2] and different redox conditions in the source. In (c), the average redox condition ($Fe^{2+}/\sum Fe$ ratio) in the source is positioned at ~0.95, depending of the titanium content of samples ($Fe_2O_3/TiO_2 = 0.5$); in (a) and (b) the redox condition in the mantle source is fixed at 0.8 and 0.9, respectively. (d) 242 samples yielded a solution of calculation when PRIMELT was running with a fixed (0.9) or Ti-dependent redox value (overlaps > 74 %). Uncertainties that arise by calculating FeO using $Fe_2O_3/TiO_2=0.5$ instead of $FeO/FeO_T=0.9$[3] propagate to uncertainties (SEM) in mantle T_P lower than 30 °C for high-Ti type lavas ($TiO_2>1$ wt% with $FeO/FeO_T <0.95$) up to 60 °C for low-Ti types ($TiO_2< 0.5$ wt% with $FeO/FeO_T >0.95$). (e) Uncertainties that arise by calculating FeO using $Fe_2O_3/TiO_2=0.5$ instead of $FeO/FeO_T=0.9$ propagate to uncertainties (SEM) in melt fraction (AFM) lower than 1% for high-Ti type lavas ($TiO_2>1$ wt% with $FeO/FeO_T <0.95$) up to 2% for low-Ti types ($TiO_2< 0.5$ wt% with $FeO/FeO_T > 0.95$).

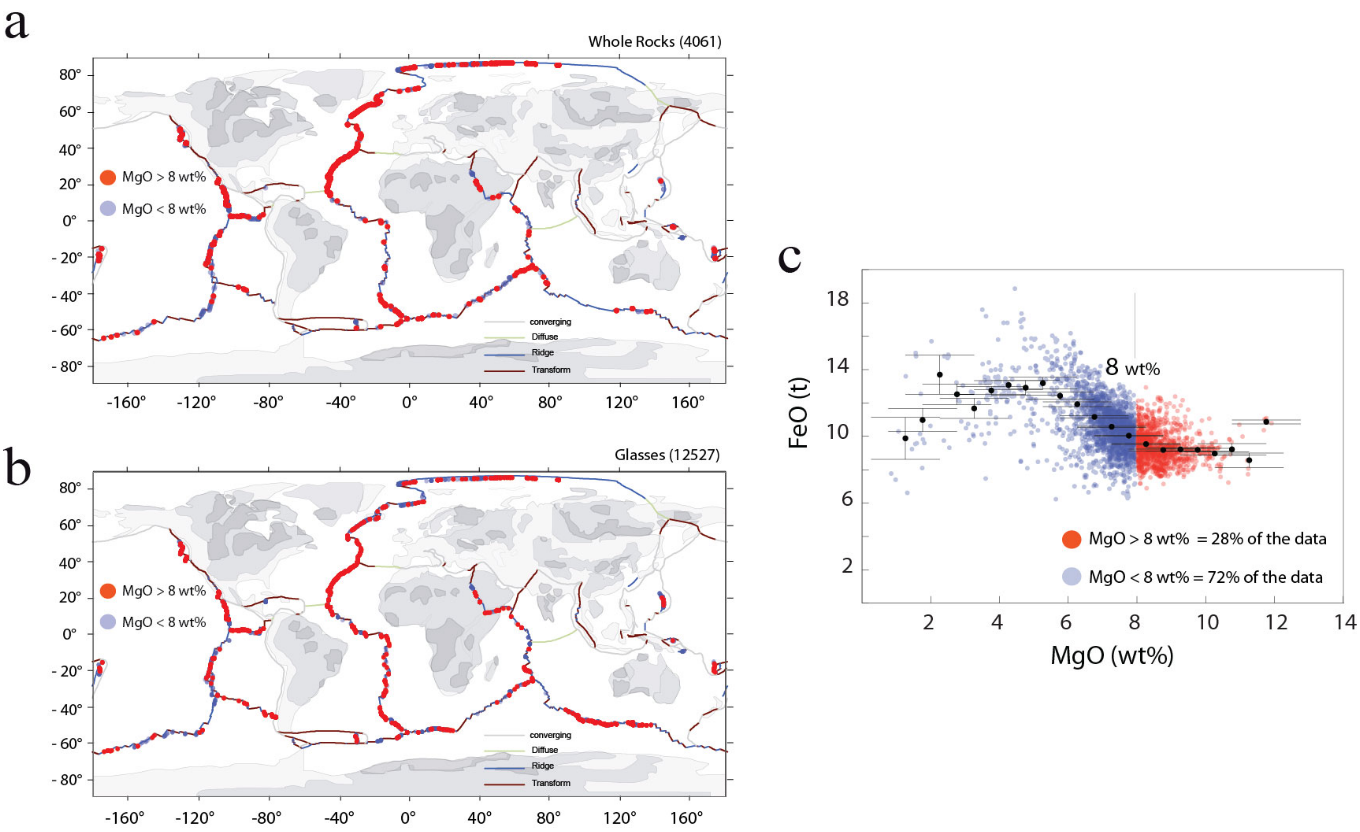

Supplementary Figure 2 – Spatial distribution of samples[1] as a function of their evolution through magma differentiation. (a) Whole-rock analyses only (25% of the dataset). (b) Glasses analyses (75% of the dataset). (c) MgO-FeO_T liquid lines of descent for MORBs. The blue points correspond to more evolved liquids where clinopyroxene fractionated, leading to an increase of FeO_T in magma with increasing differentiation (MgO decrease). A statistical assessment of their averaged evolution (black points) through magma differentiation is given by the black drawbars (uncertainty bars correspond to $\pm$ 1 σ standard deviation for a step of 0.5 wt%). Geological map contours in (a) and (b) represent Archean (dark grey), Proterozoic (grey) and Phanerozoic (light grey) terranes[4].

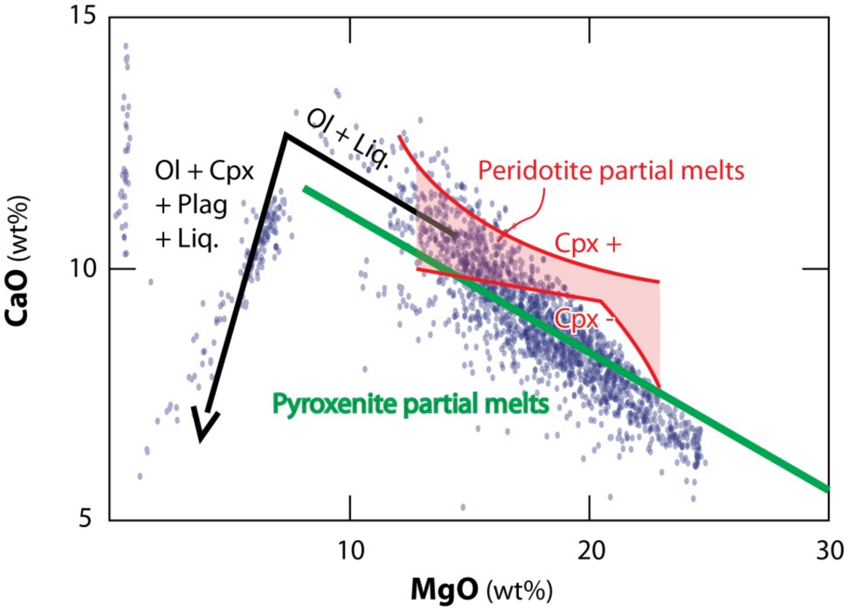

Supplementary Figure 3 - CaO and MgO contents of primary magmas calculated with PRIMELT3 software[2] using MORB compositions and reduced condition in the source ($Fe^{2+}/\sum Fe = 0.9$). Successful and unsuccessful solutions of calculation have been reported on the graph. Red lines are described by equations (9), (10), and (11) in Herzberg and Azimow, 2008[5]. They define upper and lower CaO filters of primary magmas of fertile peridotite produced by accumulated fractional melting. Primary magma with CaO contents lower than those defined by the green line are potential pyroxenite partial melts; they can also be peridotite partial melts that had clinopyroxene (Cpx) removed. The black broken arrow corresponds to the typical liquid line of descent for primary magmas that crystallize gabbro in the crust; the drop in CaO often occurs at MgO < 7–10%. However, Cpx can also crystallize in the mantle and affect magmas with MgO > 10%. Here, only ~25% of the dataset have been reported (whole-rock analyses only, see **Supplementary Figure 2a**).

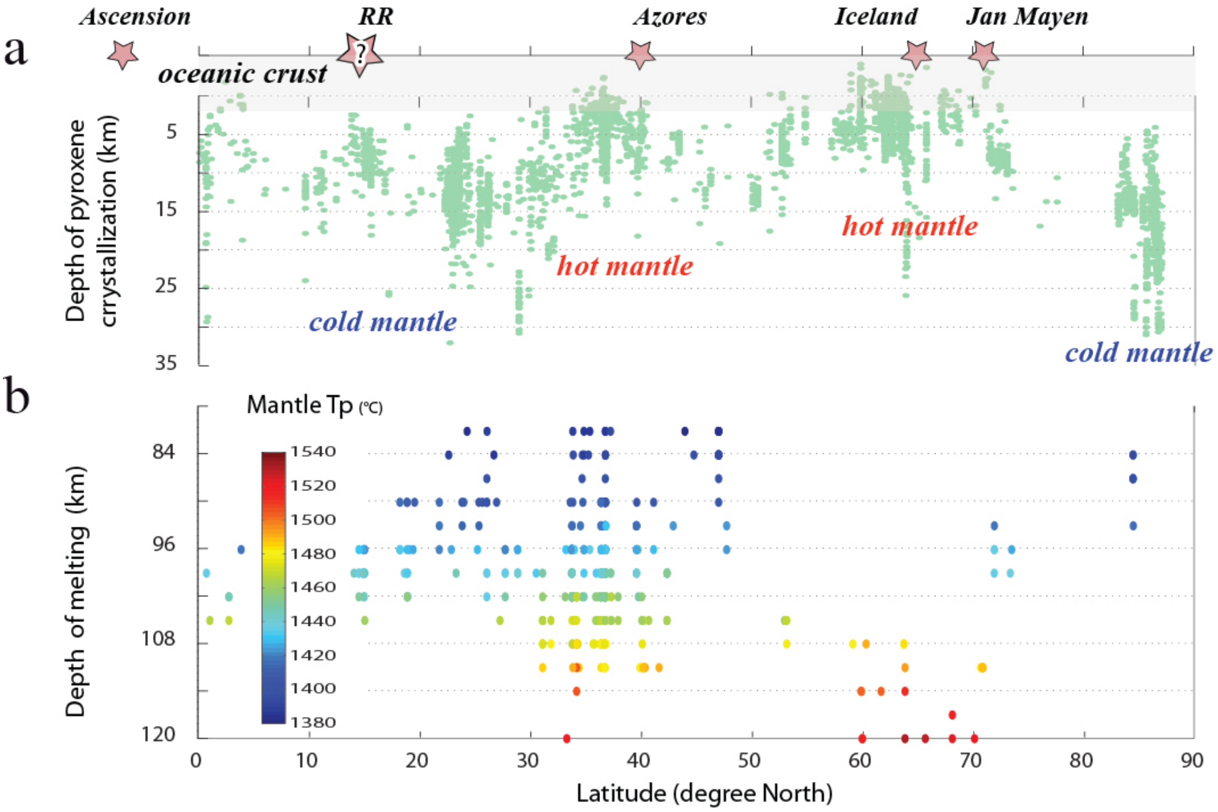

Supplementary Figure 4 – Latitudinal distribution of pyroxene crystallization depth and T_P estimates along the northern section of the MAR (a) Depth of pyroxene crystallization then subtraction (e.g. fractionation) in a primary magma that, later, will give birth to MORBs, calculated using equation (6) in Herzberg, 2004[6] and assuming a ratio of 1:3 between pressure (kb) and depth (km). (b) Depth at which adiabatically upwelling mantle below the MAR segments crossed their solidus, depending on their potential temperature (T_P) calculated with PRIMELT3 MEGA software[2] using reduced conditions ($Fe^{2+}/\sum Fe = 0.9$) in the source. Solutions of calculation have been filtered for MgO < 8 wt% (blue dots in **Supplementary Figure 2c**). The location of Jan Mayen, Iceland and Azores plume-like volcanic edifices forming on the ridge are indicated. The RR plume-like edifice corresponds to the location of the Researcher-Ridge plume, described by Long et al.[7]. The lowest pressures are observed close to Iceland where elevated T_P have been calculated. The highest pressures are observed for MORB associated with ridge segment terminations, especially segments >70°N (e.g. Gakkel ridge). Data are plotted against latitude.

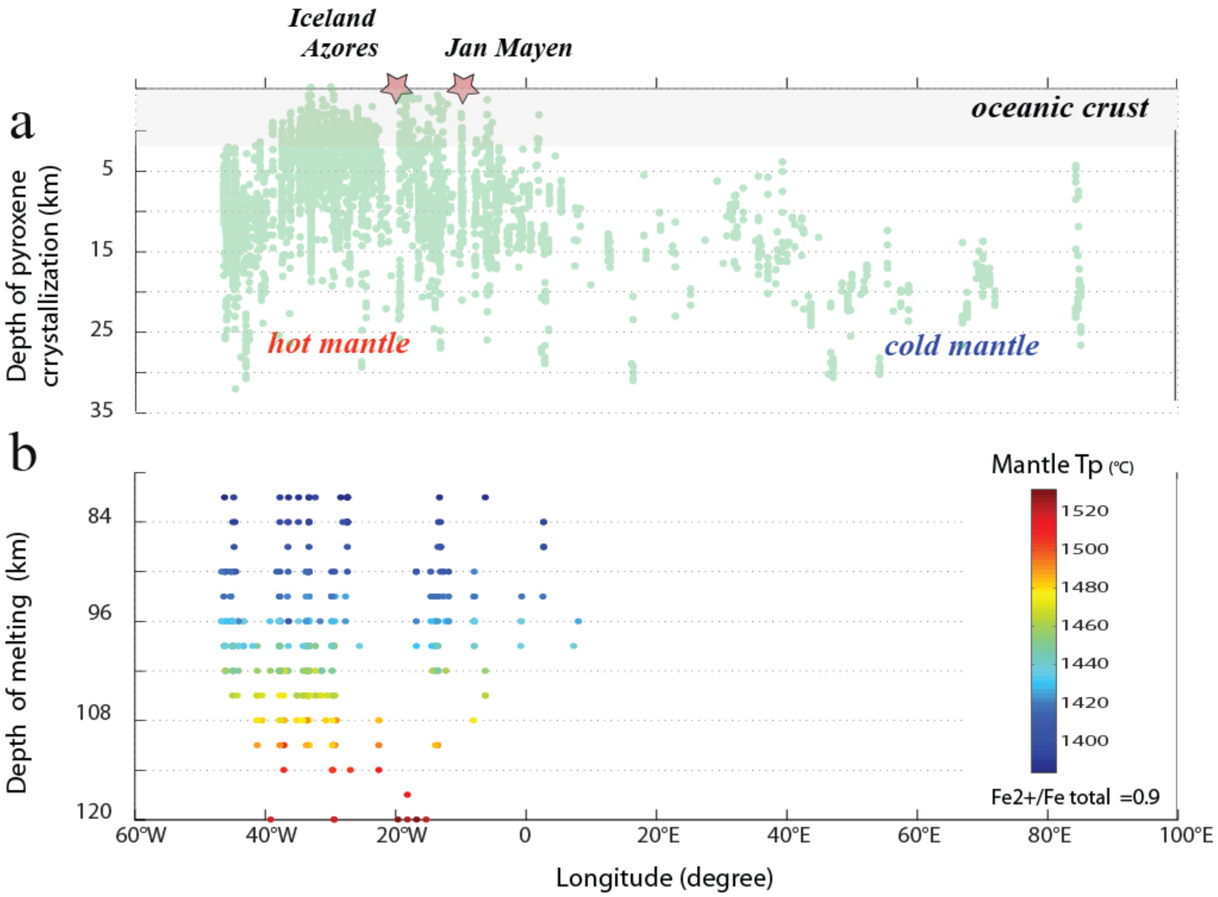

Supplementary Figure 5 – Longitudinal distribution of pyroxene crystallization depth and T_P estimates along the northern section of the MAR (a) Depth of crystallization then subtraction (e.g. fractionation) of pyroxene in a primary magma, that later will give birth to MORBs, calculated using equation (6) in Herzberg, 2004[6] and assuming a ratio 1:3 between pressure (kb) and depth (km). (b) Depth at which adiabatically upwelling mantle below the MAR segments crossed their solidus, depending on their potential temperature (Tp) calculated with PRIMELT3 Mega software[2] using reduced conditions ($Fe^{2+}/\sum Fe = 0.9$) in the source. Solutions of calculation have been filtered for MgO < 8 wt% (blue dots in **Supplementary Figure 2c**). MORBs for which Ol - Plag fractionation was likely have been filtered out as discussed in Herzberg, 2004[6] ; these have CaO > -0.3 x MgO + 14.5[6]. Stars show the location of Jan Mayen, Azores and Iceland. Data are plotted against longitude. The longitude 0° corresponds to the Greenwich meridian.

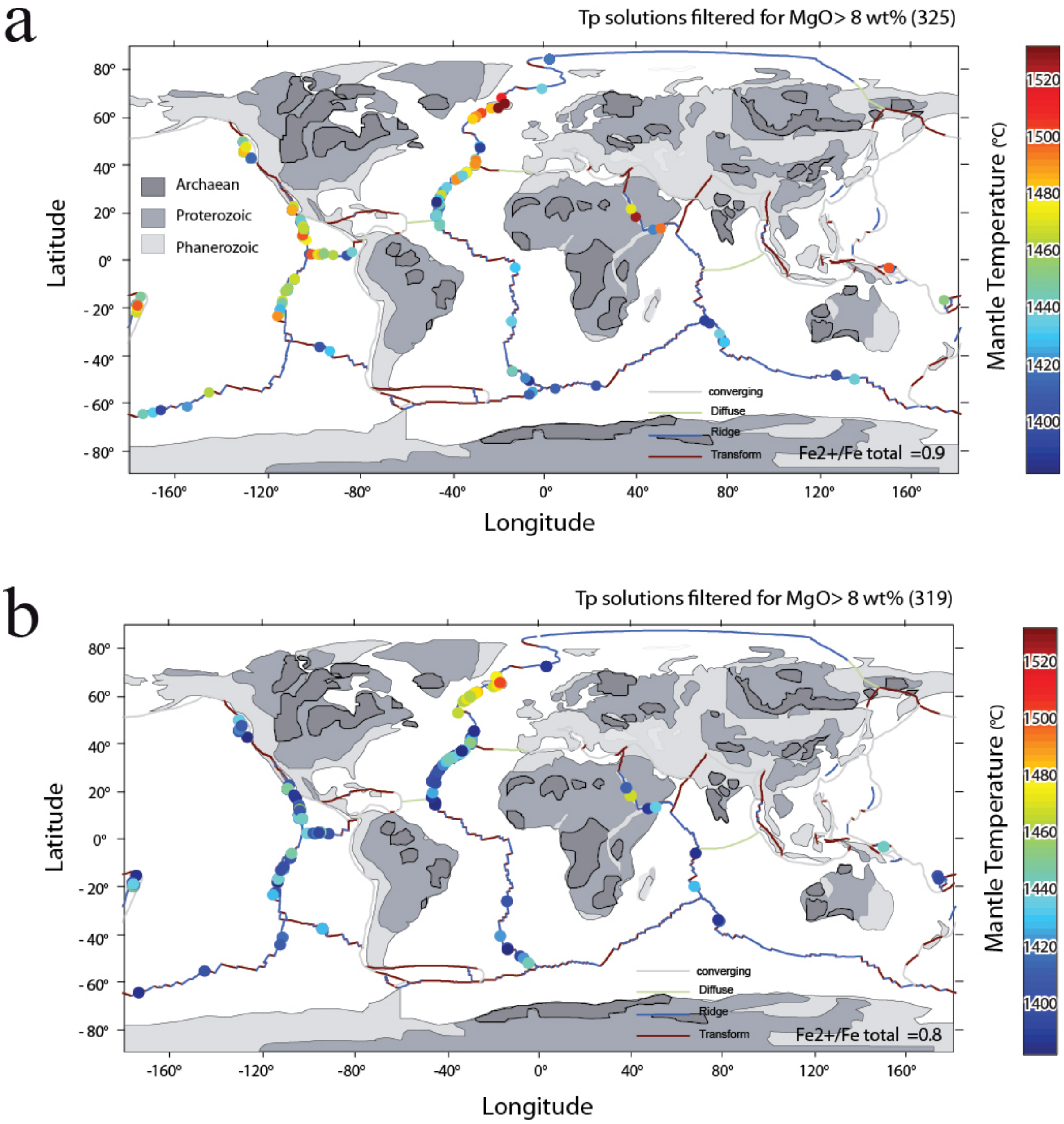

Supplementary Figure 6 – Distribution of filtered T_P solutions. Distribution of (zero-age) MORBs[1] giving a solution of calculation with PRIMELT3[2]. Mantle potential temperatures (T_P) were calculated with reduced conditions ($Fe^{2+}/\sum Fe$ = 0.9 in (a) or 0.8 in (b)) in the source and filtered with MgO < 8 wt% (blue dots in **Supplementary Figure 2c**). Here, only ~25% of the dataset have been reported (whole-rock analyses only, see **Supplementary Figure 2a**). Geological map contours represent Archean (dark grey), Proterozoic (grey) and Phanerozoic (light grey) terranes[4].

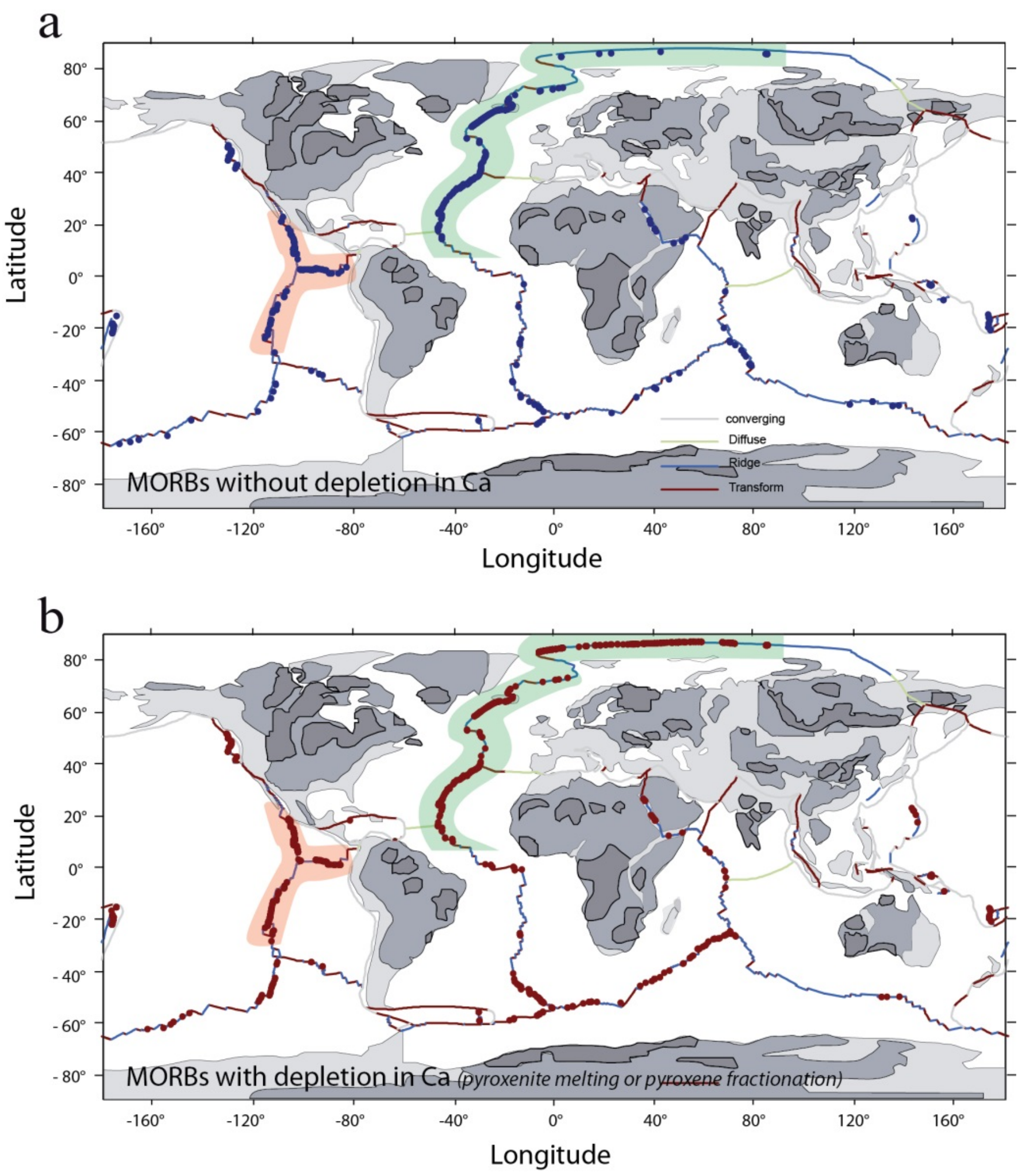

Supplementary Figure 7 – Distribution of (zero-age) MORBs[1] deriving from a primary magma (a) that is not depleted in CaO and (b) depleted in CaO. A ratio of ~32% is observed between CaO-depleted and -undepleted primary magma for the East Pacific Rise, between 30°S and 25°N (colored in orange on the map). The northern sections of the MAR (colored in green on the map) are characterized by a different ratio (57%), with significant variations along the strike of the ridge. Segments of ridges characterized by a dominant record of CaO-undepleted magma are positioned close to plume-like volcanic edifices (Iceland, Azores) whereas depleted magma are dominantly observed in ridge segment terminations, especially segments >70°N (e.g. Gakkel ridge). Here, only ~25% of the dataset have been reported (whole-rock analyses only, see **Supplementary Figure 2a**). Geological map contours represent Archean (dark grey), Proterozoic (grey) and Phanerozoic (light grey) terranes[4].

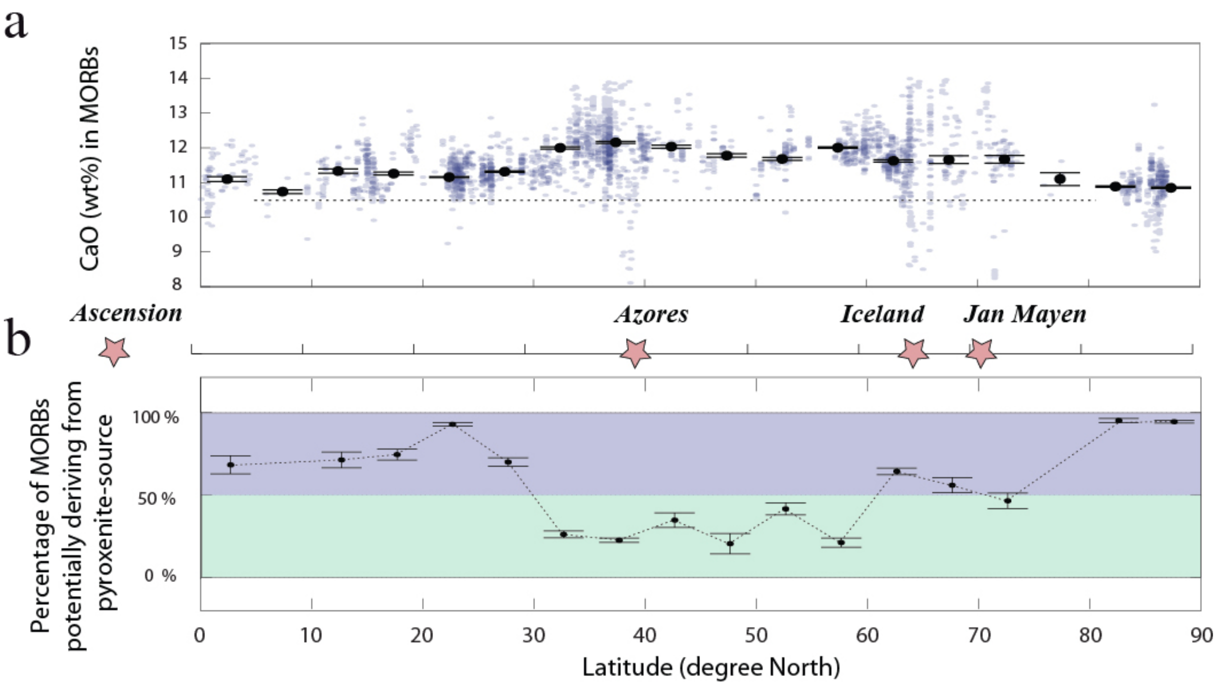

Supplementary Figure 8 – Chemical depletion in CaO along the northern section of the MAR (a) Chemical evolution of (near zero-age) MORBs sampled along the Mid-Atlantic Ridge[1]. Data have been plotted against the latitudes between 0 and 90°N along the x-axis. The Jan Mayen, Iceland and Azores plume-like volcanic edifices forming on the ridges are indicated. (b) Percentage of MORBs for which PRIMELT predicts a primary magma composition depleted in calcium. Mean values (black points), obtained by bootstrap analysis, are reported at 5° step of latitude.

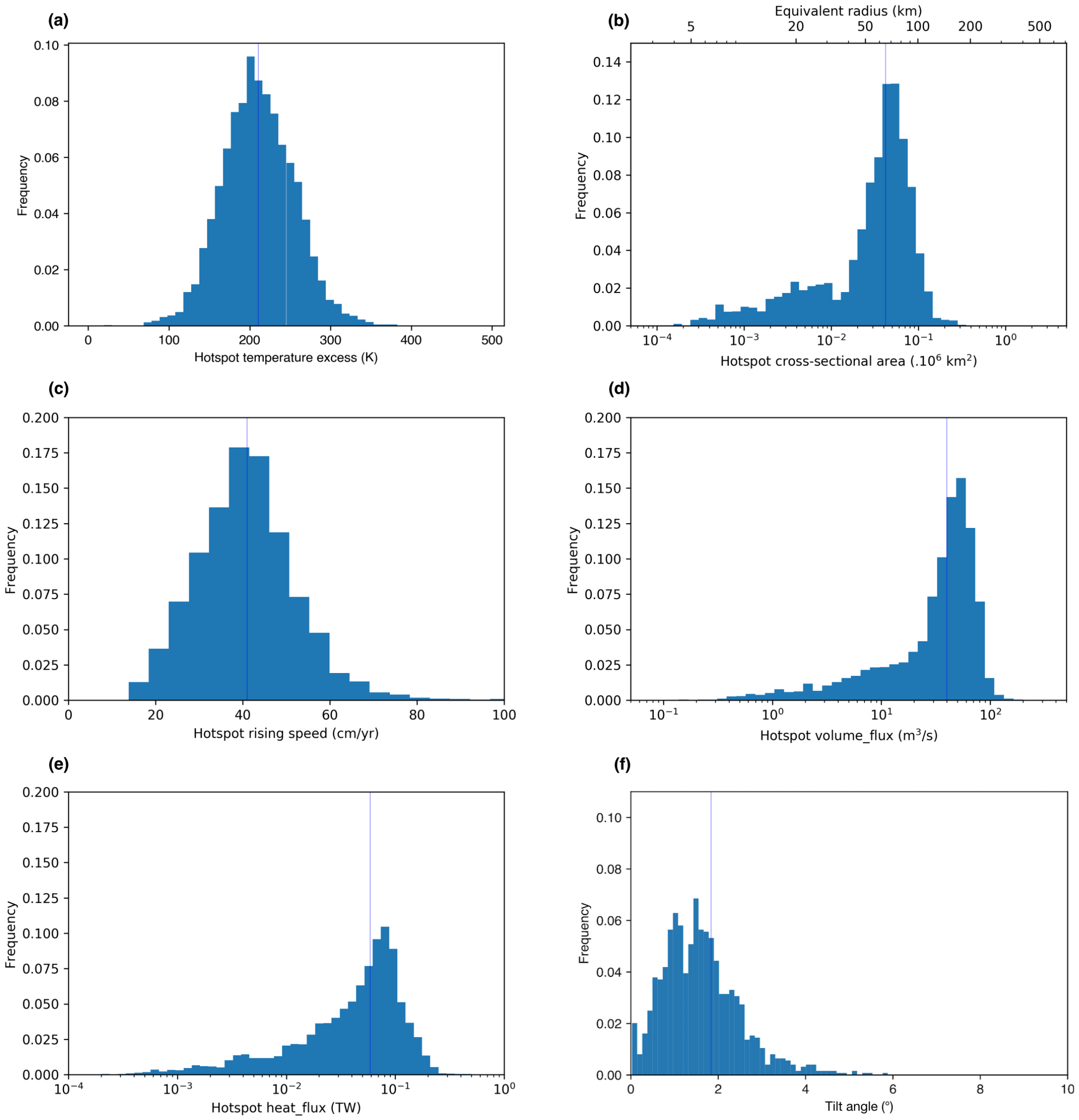

Supplementary Figure 9– Characteristics of all modelled plumes detected in the upper mantle (350 km) throughout the model time integration. Plume **(a)** temperature excess, **(b)** cross-sectional area and equivalent radius (using the approximation of a circular conduit), **(c)** rising speed, **(d)** volume flux, **(e)** heat flux and **(f)** tilt angle between the lower (1,000 km) and the upper (350 km) mantle.

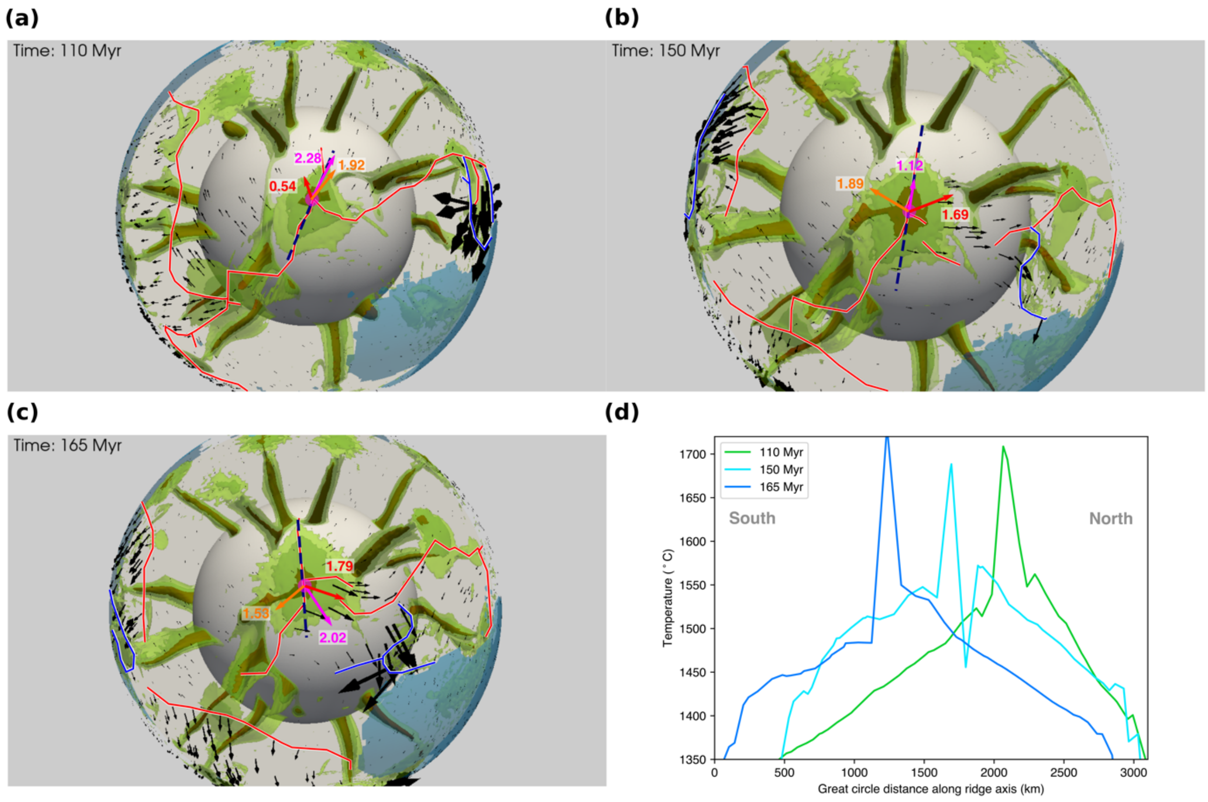

Supplementary Figure 10 – Effect of changes in the relative motion of plates and hotspot 10 on the asymmetry of the temperature profile. (a), (b) and (c) show the temporal evolution of the ridge system (red lines) and the mantle plume (light green (1,390°C), dark green (1,480°C) and brown (1,700°C) isotherms). Surface plate velocities are shown by the black arrows. Continental material is outlined in transparent blue. The average plate velocity around the considered plume is shown with the red arrow centered on the plume. The plume absolute velocity (pink arrow) is shown in magenta. Between 110 Myr and 150 Myr, the plume moves northward and its speed halves. After 150 Myr, the plume's direction changes and it moves southward at 165 Myr. At the same period of time, a subduction initiates southeast (blue lines) and propagates to the west, which leads to the reorganization of plate velocities in the vicinity of the considered plume. As a consequence, the relative velocity vector between the plume and plates rotates by 180° in 55 Myr. (d) Temporal evolution of the plume temperature profile along the ridge (corresponding to the dark blue dashed line in (a), (b) and (c)) at 100 km depth. Note the change in the symmetry of the profile through time. The corresponding symmetry coefficients are shown on **Fig. 3f**.

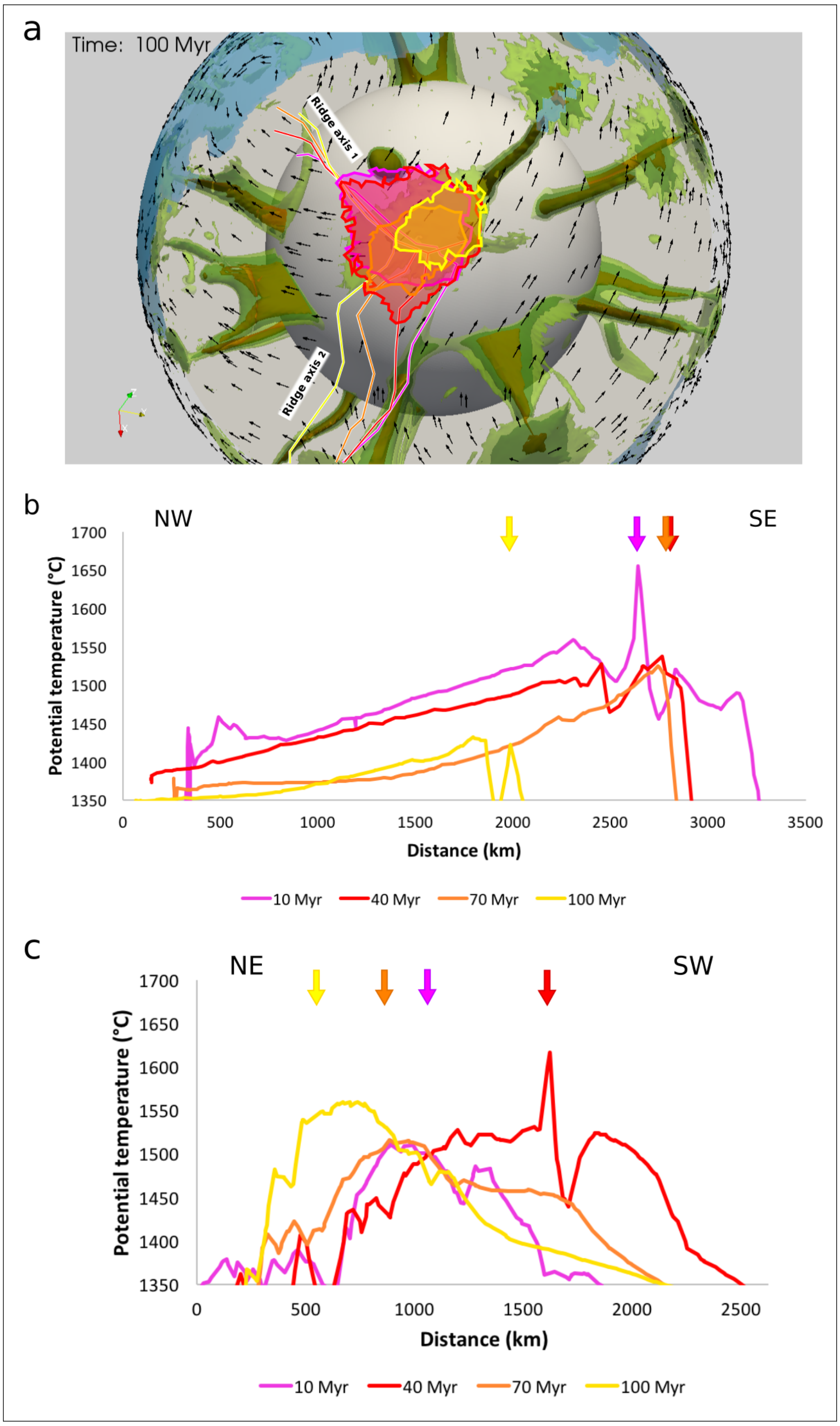

Supplementary Figure 11: Interaction of a moving plume with a moving ridge. (a) Temporal evolution of the relative position of a ridge (straight colored lines) and a mantle plume in our global model of mantle convection with plate-like behavior. Colored contour lines outline the 1480°C isotherm at 10 Myr (magenta), 40 Myr (red), 70 Myr (orange) and 100 Myr (yellow). (b-c) Temporal evolution of the potential temperature distribution at 110 km depth along axis 1 (b) and 2 (c). The colored arrows correspond to the location of the plume maximum of temperature at corresponding timesteps. Note the westward drifting ridge axis 2 and the development of an asymmetric potential temperature profile from 70 Myr along ridge axis 2.

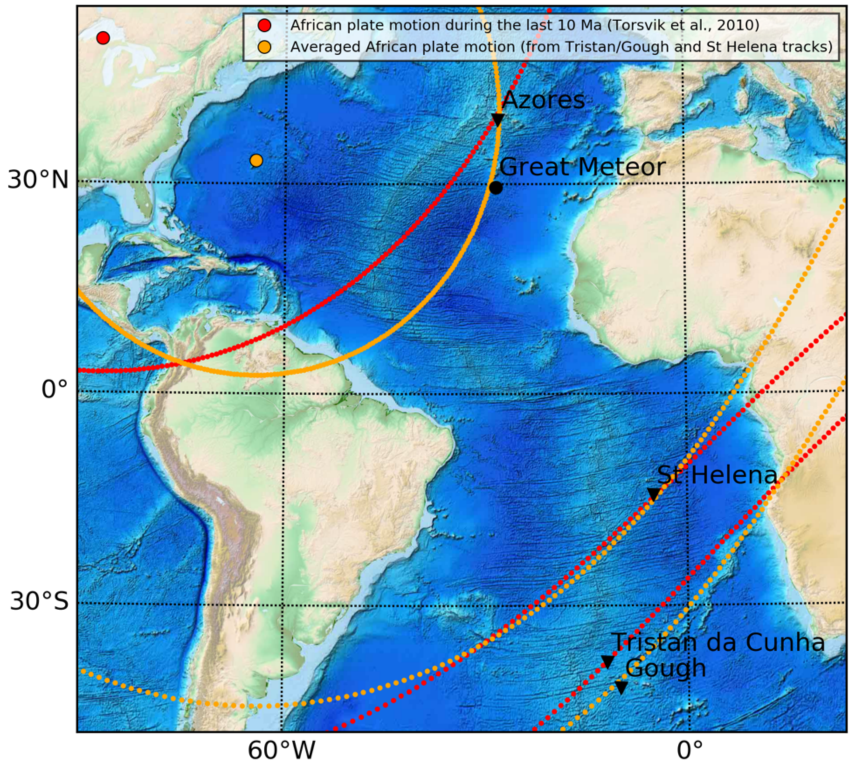

Supplementary Figure 12 – African plate motion in a reference frame relative to hotspots. The background displays the etopo map[8]. The African plate motion rotation pole from Torsvik et al., 2010[9], for the last 10 Ma is indicated by the red filled circle. Tristan da Cunha/Gough and St Helena are considered to remain stationary relative to each other[10]. The dashed red lines outline small-circles crossing the present-day position of Tristan da Cunha/Gough, St Helena and the Azores (black triangles). The orange rotation pole and small circles represent the average motion of the African plate compared to fixed Tristan da Cunha/Gough and St Helena for the last 80 Ma. The small circles fit Tristan/Gough and St Helena hotspot tracks and indicate a north-eastward rotation of Africa relatively to those hotspots. The corresponding orange small circle for the Azores present-day position superposed to the topographic high linking the Azores to Great Meteor. However, using this potential hotspot track would result in a south-westward motion of the African plate, incompatible with the tracks of Tristan/Gough and St Helena, indicating a north-eastward motion of the African plate.

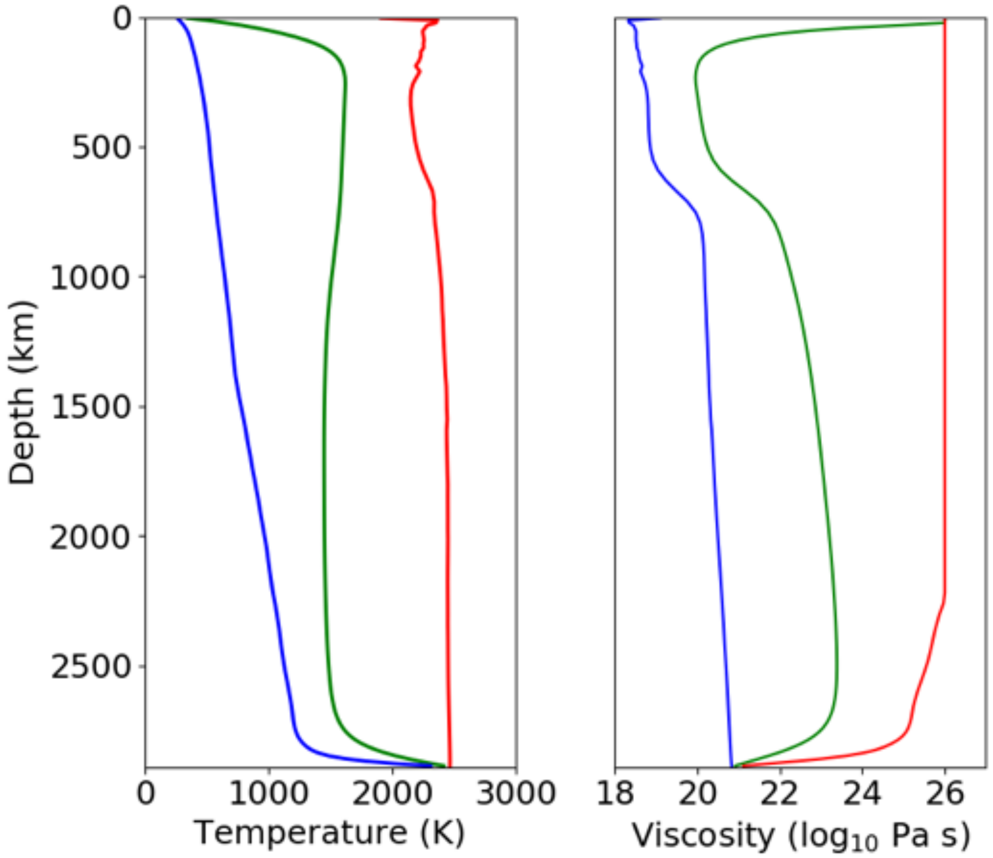

Supplementary Figure 13 –Time-averaged minimum (blue curve), mean (green curve) and maximum (red curve) geotherm (**left**) and geotherm-derived minimum (blue curve), mean (green curve) and maximum viscosity profile (**right**) of our numerical model.

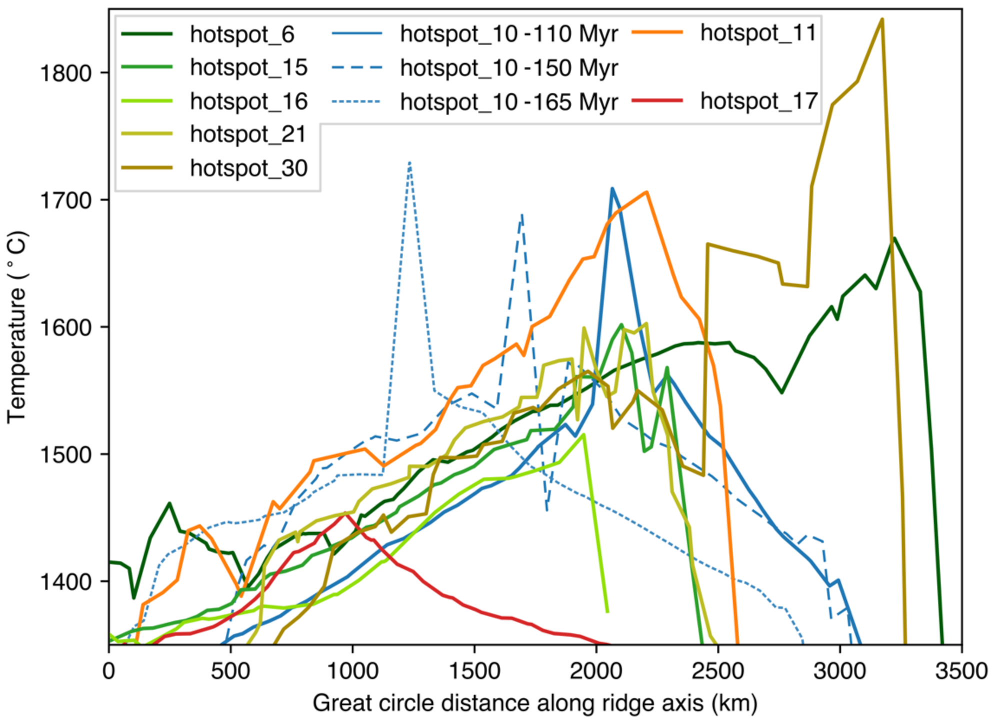

Supplementary Figure 14 – Temperature profiles at 100 km depth of the modeled plumes interacting with ridges. Hotspots 6, 15, 16, 21 and 30 display a very asymmetric profile and correspond to case 1. Hotspot 10 corresponds to case 2. The temporal evolution of its thermal asymmetry is shown (See **Supplementary Figure 11** for details). Hotspot 11's thermal profile is asymmetric and corresponds to case 3 and Hotspot 17 presents a symmetric thermal profile and corresponds to case 4. All plumes correspond to **Fig. 3f**

Supplementary Note 1:

Fig. 2a and **Supplementary Figure 8** unravel chemical changes of MORBs along the MAR. The database records slightly elevated CaO (wt%) values for MORBs centered on Azores (~40° N) and a progressive decrease further south and north of the archipelago, following a bell-shaped evolution. The percentage of samples for which PRIMELT predicts a primary magma composition depleted in calcium, by comparison with primary magmas deriving from peridotite-source, is given in **Supplementary Figure 8b**. Ca-depletion is minimal in the Azores region. It progressively increases in the south and north of the archipelago, reaching its maximum values (~100%) along the Gakkel ridge which is on the northern segment of the MAR (**Supplementary Figure 5**). Such differences in calcium cannot solely be explained by a fractionation of olivine (forsterite: Mg-rich endmember) and either require an increasingly subtraction of pyroxene (diopside : Mg_Ca-rich endmember) from the primary magma and/or an increasingly melting of Ca-depleted source (pyroxenites) in the mantle[5] which, we infer, must be related to changes in the mantle temperature below the MAR.

A cooler mantle will promote pyroxenite melting over peridotite because of its lower temperature. Likewise, low thermal gradients in a mantellic section will promote the crystallization and sequestration of pyroxene at deeper levels, decreasing the content of Ca in the residual melt. Elevated T_P values (> 1500°C) are observed in the north of the Azores archipelagos, extending to Iceland. A progressive decrease in temperature is observed southwards of the Azores and a marked decrease northwards of the Azores, giving the appearance of asymmetric thermal structure. However, a gap of data exists between 45°N and 55°N of latitude (**Supplementary Figures 6** and **7**). This section is associated with a change in the orientation of the ridge, which here operates with a strong strike-slip component (e.g. Charlie Gibbs's fault). The depth of pyroxene fractionation for MORBs was estimated using the equation (6) of Herzberg, 2004[6] and results are given in **Fig. 2a, Supplementary Figures 4a** and **5a**. MORBs in which olivine and plagioclase fractionation likely occurred have been filtered out as discussed in Herzberg, 2004[6], these have CaO > -0.3 x MgO + 14.5. As expected, lower pressures of crystallization are observed around Azores and Iceland where elevated T_P have been calculated. The pressures of crystallization increase with decreasing T_P, as observed in the south of Azores. The highest pressures are observed for MORBs associated with ridge segment terminations (e.g. Gakkel ridge).

SUPPLEMENTARY REFERENCES

1. Gale, A., Dalton, C. A., Langmuir, C. H., Su, Y. & Schilling, J.-G. The mean composition of ocean ridge basalts. *Geochemistry Geophys. Geosystems* **14,** (2013).
2. Herzberg, C. & Asimow, P. D. PRIMELT3 MEGA.XLSM software for primary magma calculation: Peridotite primary magma MgO contents from the liquidus to the solidus. *Geochemistry Geophys. Geosystems* **16,** 563–578 (2015).
3. Herzberg, C., Condie, K. & Korenaga, J. Thermal history of the Earth and its petrological expression. *Earth Planet. Sci. Lett.* **292,** 79–88 (2010).
4. Artemieva, I.M. & Mooney W.D. Thermal thickness and evolution of Precambrian lithosphere: A global study. *J. Geophys. Res.* **106**, 16387-16414 (2001).
5. Herzberg, C. & Asimow, P. D. Petrology of some oceanic island basalts: PRIMELT2.XLS software for primary magma calculation. *Geochemistry Geophys. Geosystems* **9,** (2008).
6. Herzberg, C. Partial crystallization of mid-ocean ridge basalts in the crust and mantle. *J. Petrol.* **45,** 2389–2405 (2004).
7. Long, X., Geldmacher, J., Hoernle, K., Hauff, F., Wartho, J. A., Garbe-Schönberg, D., & Grevemeyer, I. Age and origin of Researcher Ridge and an explanation for the 14° N anomaly on the Mid-Atlantic Ridge by plume-ridge interaction. *Lithos*, **326**, 540-555 (2019).
8. Amante, C. & Eakins, B. W. ETOPO1 1 arc-minute global relief model: procedures, data sources and analysis. *NOAA Tech. Memo.* (2009). doi:10.1594/PANGAEA.769615
9. Torsvik, T. H., Steinberger, B., Gurnis, M. & Gaina, C. Plate tectonics and net lithosphere rotation over the past 150 My. *Earth Planet. Sci. Lett.* **291,** 106–112 (2010).
10. Duncan, R. A. Hotspots in the southern oceans - an absolute frame of reference for motion of the Gondwana continents. *Tectonophysics* **74,** 29–42 (1981).
