# Supplementary material for: Northward drift of the Azores plume in the Earth’s mantle
Source: Nat Commun. 2019 Jul 19;10:3235. doi: 10.1038/s41467-019-11127-7 (PMC6642178; doi:10.1038/s41467-019-11127-7)
Supplement: Supplementary file 3 — Description of Additional Supplementary Files [file 41467_2019_11127_MOESM3_ESM.pdf]

Description of Additional Supplementary Files

File Name: Supplementary Data 1
Description: **Solutions of our calculation for all samples used in this study. Table 1a** shows the MORBs compositions along the MAR, their mantle origin (peridotite vs pyroxenite) and the pressures of crystallization of magmatic pyroxenes (fractionation). **Table 1b** shows the calculation results from PRIMELT.
